# Commentary: Autoimmune diseases in patients with myotonic dystrophy type 2

**DOI:** 10.3389/fneur.2022.1041437

**Published:** 2022-11-17

**Authors:** Manon J. Damen, Alfons A. den Broeder, Nicol C. Voermans, Alide A. Tieleman

**Affiliations:** ^1^Department of Neurology, Donders Institute for Brain, Cognition and Behaviour, Radboud University Medical Center, Nijmegen, Netherlands; ^2^Department of Neurology, Medisch Spectrum Twente, Enschede, Netherlands; ^3^Department of Rheumatology, Sint Maartenskliniek, Nijmegen, Netherlands

**Keywords:** T cell immunity, B cell immunity, interleukin 6 (IL-6), interleukin 12 (IL-12), Th1, CCHC-Type Zinc Finger Nucleic Acid Binding Protein (CNBP), myotonic dystrophy type 2 (DM2), autoimmune diseases (AIDs)

## Introduction

With interest, we read the article by Peric et al. about the frequency and type of autoimmune diseases (AIDs) in a large cohort of Serbian patients with myotonic dystrophy type 2 (DM2) (1). Their results are in line with the previously high incidence of autoimmune diseases in DM2 reported in the Dutch cohort (2).

In the conclusion of their article, however, the authors state that “*The pathogenesis of AIDs in DM2 remains an intriguing question*” and “*To understand better the autoimmunity in DM2, future studies should also focus on assays to measure B cell and T cell activity and interleukin pathways*.” We would like to comment on these statements below based on our previous observations and recent immunological research in DM2.

## Discussion

Peric et al. described a high frequency (28.8%, *n* = 36/125) of AIDs in the Serbian cohort of patients with DM2 (1), a finding that is very similar to the observations in our 2009 study on AIDs (21.4%, *n* = 6/28, compared to 2.0% in a DM1 control group) (2, 3). We also proposed possible explanations for the association between DM2 and AIDs.

**Figure 1:**
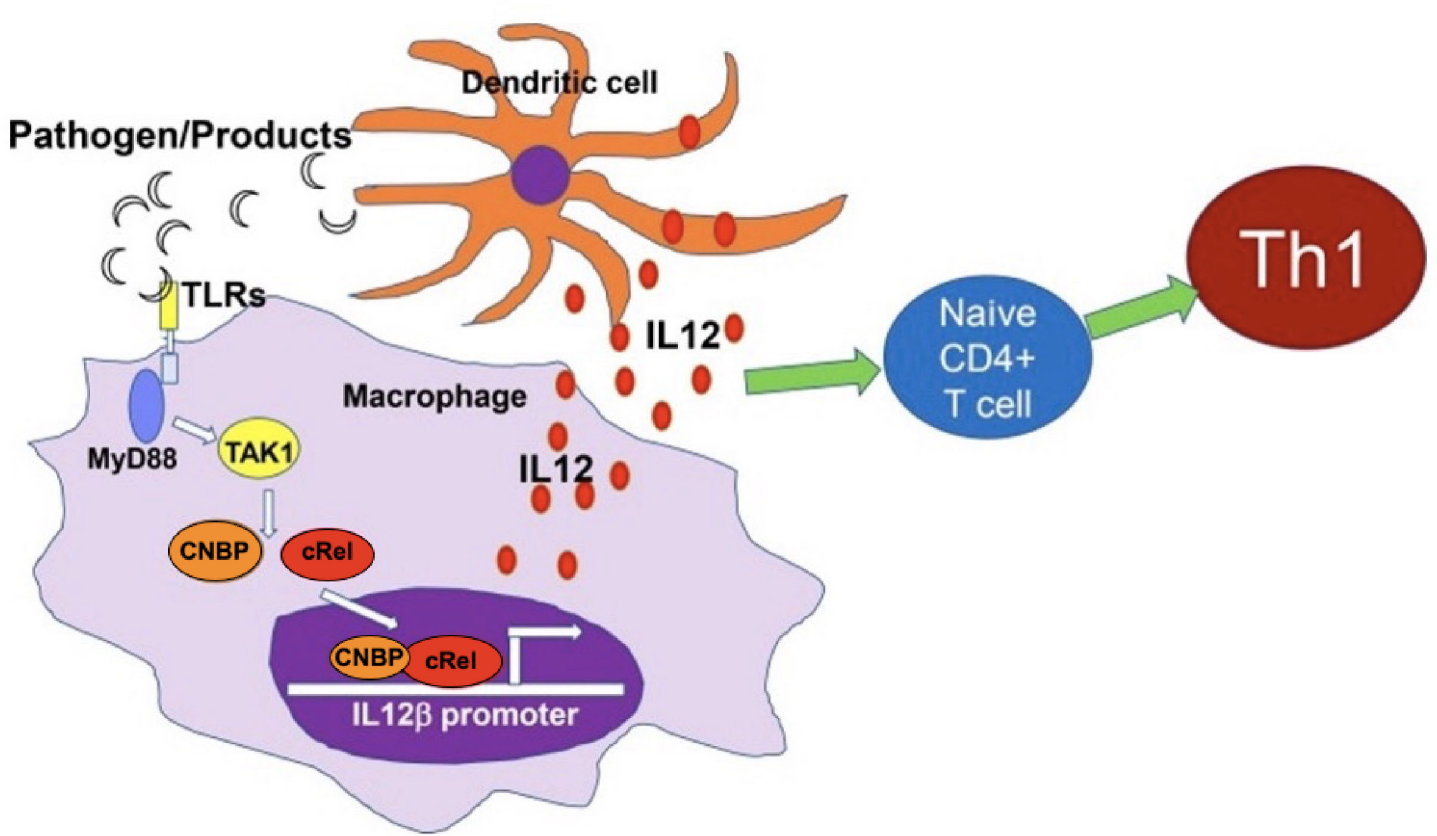
CNBP regulates IL-12β gene transcription, IL-12 production, and Th1 immunity. CNBP resides in the cytosol of macrophages and translocates to the nucleus in response to diverse microbial pathogens and pathogen-derived products through the TLR-MyD88-IRAK-TAK1 signaling pathway. CNBP has a selective ability to control the activation of c-Rel, a key driver of IL-12β gene transcription. The nuclear translocation and DNA binding activity of c-Rel require CNBP. Furthermore, CNBP itself binds the IL-12β promoter. Figure reprinted and adapted with permission of the author and publisher and used under CC BY-NC-SA (4).

In the last decade, immunological studies both *in vitro* and *in vivo* (CCHC-Type Zinc Finger Nucleic Acid Binding Protein (CNBP) depleted zebrafish, CNBP-deficient mice, and human blood samples) revealed important roles of CNBP as a novel transcription regulator of interleukin-12β (IL-12β) gene transcription in macrophages and in IL-12-driven and Th1-mediated immune responses (Figure 1) (4, 5). CNBP has also been shown to be a transcriptional regulator of IL-6 in inflammatory responses (6). Furthermore, the analysis of expression of CNBP in normal human tissues revealed that immune cells express this gene the most, particularly B and T lymphocytes (7).

DM2 is caused by a tetranucleotide (CCTG) repeat expansion in intron 1 of the CNBP gene resulting in both an RNA gain-of-function and a CNBP loss of function. We, therefore, consider the results of the recent immunological studies summarized above very relevant for the observations in the Serbian cohort (8). The studies reveal that CNBP contributes to the coordination of immune gene expression by regulating IL-12β and IL-6 gene transcription, IL-12 production, and Th1 immunity. This likely creates a pro-inflammatory state, and therefore, a higher incidence of AIDs in DM2.

In conclusion, CNBP has recently been identified as a key transcriptional regulator required for activating and maintaining the immune response. This role of CNBP suggests an additional explanation in the pathogenesis of the wide variety of AIDs in DM2.

## Author contributions

MD wrote the first draft of the manuscript. AT wrote sections of the manuscript. MD, AB, NV, and AT contributed to the manuscript revision and read and approved the submitted version.

## Conflict of interest

The authors declare that the research was conducted in the absence of any commercial or financial relationships that could be construed as a potential conflict of interest.

## Publisher's note

All claims expressed in this article are solely those of the authors and do not necessarily represent those of their affiliated organizations, or those of the publisher, the editors and the reviewers. Any product that may be evaluated in this article, or claim that may be made by its manufacturer, is not guaranteed or endorsed by the publisher.
